# Exploring the relationships between electronic blackmail awareness, smartphone addiction, and escapism among nursing students: a structural equation modeling approach

**DOI:** 10.1186/s12912-026-04710-z

**Published:** 2026-05-11

**Authors:** Samia Ibrahim Mabrouk Baraka, Samia Mohamed Sobhi Mohamed, Nagwa Ibrahim Mabrouk Baraka

**Affiliations:** 1 Community Health Nursing, Faculty of Nursing, Tanta University, Tanta, Egypt; 2 Nursing Administration, Faculty of Nursing, Alexandria University, Alexandria, Egypt; 3 Pediatric Nursing, Faculty of Nursing, Tanta University, Tanta, Egypt

**Keywords:** Cybercrime, Escapism, Internet use, Nursing students, Smartphone addiction, Structural equation modeling

## Abstract

**Background:**

Digital threats including smartphone addiction, escapist tendencies, and electronic blackmail are becoming increasingly prevalent among nursing students. While electronic blackmail awareness is crucial, it is yet unknown how it affects behavioral results. This study looked at how nursing students’ knowledge of electronic blackmail and their desire for escape are mediated by smartphone addiction.

**Methods:**

A quantitative, cross-sectional, correlational design using Structural Equation Modeling (SEM) was conducted in accordance with STROBE guidelines. A sample of 440 undergraduate nursing students from the Faculty of Nursing, Tanta University, Egypt, was recruited using a stratified systematic sampling approach. Data was collected using three distinct instruments which assess electronic blackmail awareness through a knowledge-based assessment, smartphone addiction, and escapism.

**Results:**

The mean age of the participants was 20.57 years (SD = 1.30). Most participants were female (69.8%) and from rural areas (60.9%). Approximately half reported insufficient family income (51.6%). Structural path analysis showed that electronic blackmail awareness did not significantly predict smartphone addiction (B = 0.035, β = 0.034, *p* = 0.469) or escapism (B = 0.001, β = 0.001, *p* = 0.996). In contrast, smartphone addiction demonstrated a strong and statistically significant positive association with escapism (B = 0.645, β = 0.664, *p* < 0.001). Model fit indices indicated acceptable model fit (χ²/df = 3.112, NFI = 0.918, RFI = 0.901, IFI = 0.943, CFI = 0.942, RMSEA = 0.061).

**Conclusions:**

Although awareness of electronic blackmail was not significantly associated with escapism among nursing students, smartphone addiction emerged as a strong and significant predictor of escapist behavior. These findings suggest smartphone addiction was significantly associated with escapism and may represent an important behavioral factor related to maladaptive coping patterns among nursing students. Addressing smartphone addiction through targeted interventions, such as promoting responsible smartphone use, strengthening psychosocial support, and enhancing digital resilience, may help reduce escapist behaviors and support students’ academic and psychological well-being. In addition, integrating awareness programs related to cyber risks, including electronic blackmail, may further contribute to improving students’ digital safety and coping strategies.

**Clinical trial number:**

Not applicable.

**Supplementary Information:**

The online version contains supplementary material available at 10.1186/s12912-026-04710-z.

## Introduction

The pervasive use of smartphones in daily life has significantly influenced nursing students’ behaviors and overall well-being. Smartphones provide convenient access to educational resources, communication tools, and social networking platforms; however, excessive use has raised concerns regarding problematic smartphone use and addiction among university students. A recent longitudinal study involving 637 nursing undergraduates reported that more than half of the participants exhibited moderate to severe levels of smartphone addiction, which was associated with poorer academic performance, increased anxiety, and irregular sleep patterns [1]. Students facing demanding academic and clinical training requirements may be particularly vulnerable to addictive smartphone use, characterized by compulsive checking behaviors, tolerance, and withdrawal symptoms when access to the device is restricted. Such patterns may impair students’ ability to manage stress effectively and maintain professional standards in clinical environments [2].

At the same time, university students are increasingly exposed to cyber risks, including electronic blackmail, sometimes referred to as cyber extortion or sextortion when it involves threats to disclose private images or information. Despite growing concerns about this form of cybercrime, the mechanisms, prevalence, and psychological consequences of electronic blackmail remain insufficiently understood. Existing studies indicate that more than half of surveyed university students reported either personal or indirect exposure to electronic blackmail incidents [3]. Although increased awareness of cyber threats may improve risk recognition and preventive behaviors, it may also heighten perceived vulnerability and psychological distress. This heightened perception of risk may lead students to adopt avoidant coping strategies, particularly when they feel uncertain about how to respond to potential cyber threats.

One such coping behavior is escapism, defined as engaging in activities that divert attention from stress, negative emotions, or challenging life situations. In digital contexts, escapism often involves excessive engagement with smartphones and online platforms as a means of temporarily avoiding academic or psychological stress. Previous research has demonstrated that escapism can mediate the relationship between academic stress and problematic smartphone use, with students who exhibit stronger escapist tendencies engaging in higher levels of excessive smartphone use [4]. Furthermore, smartphone addiction has been identified as a significant predictor of psychological escapism, explaining a considerable proportion of the variance in escapist behaviors among university students [4].

A growing body of literature suggests that smartphone addiction may function as a behavioral mechanism linking psychological vulnerabilities to maladaptive coping behaviors. For instance, Barakat (2022) reported that smartphone addiction mediated the relationship between low self-efficacy and escapism, indicating that students with reduced confidence tended to rely on excessive smartphone use as a form of avoidance-oriented coping [5]. Similarly, Zhang et al. (2024) demonstrated that smartphone addiction acts as a pathway through which academic stress contributes to psychological distress, with sleep problems and mobile phone addiction jointly mediating the relationship between academic stress and depressive symptoms [6]. Moreover, Wang et al. (2022) found that smartphone addiction partially mediated the associations between emotional dysregulation and negative behavioral outcomes, including loneliness, sadness, and disordered eating behaviors [7]. Collectively, these findings highlight the role of smartphone addiction as an important behavioral mechanism connecting psychological stressors with maladaptive coping patterns.

Despite the growing body of research on smartphone addiction and escapism among university students, the interaction between cyber-risk awareness and maladaptive technology-related coping behaviors remains insufficiently explored, particularly among nursing students who face substantial academic and clinical demands. Previous studies have largely examined smartphone addiction because of psychological stressors such as academic pressure, emotional dysregulation, or low self-efficacy [4–7]. Likewise, research on electronic blackmail has primarily focused on prevalence, risk factors, and victimization experiences, with limited attention given to how awareness of cyber threats may influence students’ behavioral responses or coping strategies [3]. Consequently, little empirical evidence exists regarding whether awareness of cyber threats influences students’ smartphone use behaviors or their tendency to engage in escapism. Addressing this gap is particularly important in nursing education, where students must cope with demanding workloads while maintaining psychological resilience and professional competence.

## Significance of study

By addressing this gap, the present study contributes to the growing literature on nursing education, cyber psychology, and digital well-being. While previous research has investigated smartphone addiction and escapism separately [4, 5] and explored the psychological impact of cyber-victimization awareness [3], the current study integrates these constructs within a unified conceptual framework relevant to nursing students. This integrative approach enhances understanding of how digital stressors and cyber-risk awareness may influence maladaptive coping behaviors, including whether awareness of electronic blackmail unintentionally contributes to excessive smartphone use and escapism.

Recent applications of latent-variable modeling techniques, such as structural equation modeling (SEM), have demonstrated their value in examining complex relationships among behavioral and psychological constructs [8, 9]. Building on these methodological advances, the present study employs SEM to explore both direct and indirect relationships among electronic blackmail awareness, smartphone addiction, and escapism. The findings may provide important empirical evidence to inform targeted interventions within nursing education.

If smartphone addiction plays a mediating role in these relationships, nursing education programs could incorporate digital-wellness initiatives, including workshops on responsible smartphone use, cognitive-behavioral strategies to reduce compulsive checking behaviors, and practical time-management skills that promote healthier digital habits [4, 5]. In addition, integrating cyber-safety and electronic blackmail awareness modules into academic and clinical curricula may help students recognize digital threats, respond appropriately, and avoid fear-based or avoidant coping strategies. Such interventions align with broader efforts to promote psychological resilience and professional responsibility among nursing students [10]. Because the ability of nursing students to manage digital pressures is essential for academic success, clinical performance, and patient safety, identifying factors that contribute to maladaptive escapism can support the development of educational policies and well-being programs aimed at fostering a technically competent and psychologically resilient nursing workforce [1, 2].

## Research hypotheses

**Fig. 1:**
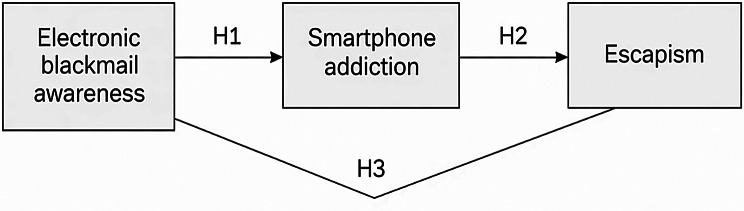
Conceptual model of the hypothesized relationships among electronic blackmail awareness, smartphone addiction, and psychological escapism

To fill this gap, the current study focuses on the direct and indirect relationships between smartphone addiction, escapism, and knowledge of electronic blackmail. The approach initially assesses direct routes, such as how awareness of electronic blackmail affects smartphone addiction and escapism and how smartphone addiction affects escapism. The study next investigates a mediated pathway in which awareness of electronic blackmail affects escape through smartphone addiction acting as a behavioral mechanism. This dual approach enables the model to investigate whether awareness influences psychological coping strategies directly or indirectly through behavioral patterns associated with technology. The following hypotheses were proposed for this study as shown in Fig. 1:

### Direct effects

#### H1

Electronic blackmail awareness is associated with psychological escapism among nursing students.

#### H2

Electronic blackmail awareness is associated with smartphone addiction among nursing students.

#### H3

Smartphone addiction is positively associated with psychological escapism among nursing students.

### Mediated effect

#### H4

Smartphone addiction was examined as a potential indirect pathway in the relationship between electronic blackmail awareness and psychological escapism among nursing students.

## Study design and setting

This study used a quantitative, cross-sectional, correlational research design based on Structural Equation Modeling (SEM) to investigate the relationships between undergraduate nursing students’ awareness of electronic blackmail, smartphone addiction, and psychological escapism. Although SEM is frequently associated with causal modeling, its usage in cross-sectional research is well-established for evaluating both direct and indirect associations concurrently, validating theoretically developed path models, and analyzing complex associative structures (Kline, 2015). As a result, rather than having causal effects, all path estimates in this study are recognized as associations. Data were collected using a structured, self-administered questionnaire consisting of standardized and validated Likert-scale items measuring each study construct. The proposed model allowed examination of both direct and indirect relationships among variables. The study was conducted at the Faculty of Nursing, Tanta University. The faculty is one of the largest nursing education institutions in the region, offering undergraduate and graduate nursing programs. It provides a diverse academic environment supported by lecture halls, clinical skill laboratories, and partnerships with teaching hospitals. This setting facilitated access to a large and diverse population of nursing students, enabling the collection of reliable and representative data.

The target population consisted of all undergraduate nursing students enrolled in the Faculty of Nursing at Tanta University during the academic year of data collection (*N* = 4,211). Eligible participants included undergraduate nursing students from all academic levels (first to fourth year) who were willing to participate voluntarily in the study and were able to read and complete the questionnaire independently. Postgraduate students and those who were absent during the data collection period were excluded. In addition, questionnaires with substantial missing responses (greater than 20% incomplete items) were excluded from the final analysis to ensure data quality and accuracy.

Sample size adequacy for Structural Equation Modeling was determined based on established methodological recommendations. SEM studies typically require large samples to ensure stable parameter estimation and adequate statistical power. According to methodological guidelines, achieving statistical power of 0.80 at an alpha level of 0.05 when evaluating model fit using the Root Mean Square Error of Approximation (RMSEA) generally requires 300–400 participants, particularly for models with moderate complexity and approximately 50 degrees of freedom [11].In addition, SEM literature recommends maintaining a ratio of 10–20 participants per estimated parameter to ensure reliable estimation and model stability [12]. The proposed structural model in this study included approximately 20–25 parameters, including latent constructs, factor loadings, structural paths, and error variances. Accordingly, the recommended sample size ranged between 200 and 500 participants.

Based on these methodological recommendations and considering the inclusion of mediating pathways, a target sample size of 400 participants was determined to provide adequate statistical power and robust model estimation. To ensure adequate representativeness, proportional stratified sampling was employed across all academic levels, as shown in Table 1. The total population consisted of 4,211 nursing students distributed across four levels. The number of students selected from each level was calculated using the following formula:

$$\begin{aligned}\:\mathrm{S}\mathrm{a}\mathrm{m}\mathrm{p}\mathrm{l}\mathrm{e}\:\mathrm{f}\mathrm{r}\mathrm{o}\mathrm{m}\:\mathrm{L}\mathrm{e}\mathrm{v}\mathrm{e}\mathrm{l}=&\left(\frac{\mathrm{T}\mathrm{o}\mathrm{t}\mathrm{a}\mathrm{l}\:\mathrm{S}\mathrm{t}\mathrm{u}\mathrm{d}\mathrm{e}\mathrm{n}\mathrm{t}\mathrm{s}\:\mathrm{i}\mathrm{n}\:\mathrm{L}\mathrm{e}\mathrm{v}\mathrm{e}\mathrm{l}}{\mathrm{T}\mathrm{o}\mathrm{t}\mathrm{a}\mathrm{l}\:\mathrm{P}\mathrm{o}\mathrm{p}\mathrm{u}\mathrm{l}\mathrm{a}\mathrm{t}\mathrm{i}\mathrm{o}\mathrm{n}}\right)\\&\times\:\mathrm{T}\mathrm{o}\mathrm{t}\mathrm{a}\mathrm{l}\:\mathrm{S}\mathrm{a}\mathrm{m}\mathrm{p}\mathrm{l}\mathrm{e}\:\mathrm{S}\mathrm{i}\mathrm{z}\mathrm{e}\end{aligned}$$

Using this formula, proportional allocation yielded 103 students from the first level (out of 1,080), 98 from the second (out of 1,030), 102 from the third (out of 1,070), and 97 from the fourth (out of 1,031), giving a total of 400 participants. Within each stratum, systematic random sampling was then applied. The sampling interval was calculated by dividing the total number of students at each level by the required sample size (e.g., 1,080 ÷ 103 ≈ 10 for the first level). A random starting point was chosen within the first interval, and every tenth student was selected until the quota was reached. To account for possible non-responses or incomplete questionnaires, a 10% attrition rate was added, increasing the final sample size to 440 participants. This ensured that the study met the recommended SEM thresholds, providing adequate statistical power, stable model estimates, and generalizable results.

### Tools for data collection

Three different tools were utilized to collect the required data, which include the following.

### Tool I: Students’ social media usage assessment questionnaire

Sampasa-Kanyinga et al. (2019) & Al Habsi et al. (2021) [13, 14] are the foundation of the tool created by El Sharkawy et al. (2024) [15] to evaluate students’ sociodemographic traits as well as comprehension of electronic blackmail. It is made up of three sections: Section I: Sociodemographic information about school-age children, including age, gender, education, place of residence, and family income. Social media usage in Part II Whether or not to use social media, how to access applications, popular platforms, daily time spent, relationship with parents, parental supervision on social media, parental discussion of electronic blackmail and prevention, parental need for educational programs, prior faculty workshops, and prior exposure to electronic blackmail were all covered in this section. These data were collected to provide contextual information about participants’ digital engagement environment. Part III assessed electronic blackmail awareness through a knowledge-based assessment consisting of 13 multiple-choice questions (MCQs). The items covered several aspects related to electronic blackmail, including the benefits and uses of social media, the concept of electronic blackmail (definition, causes or motives, and types), the hotline for reporting electronic crimes, appropriate response strategies, preventive measures, and the role of faculty in educating students about electronic blackmail. Each item was scored as 2 for a complete and correct response, 1 for an incomplete response, and 0 for an incorrect or uncertain response. The total score was calculated by summing all item responses and was treated as a continuous variable representing the level of electronic blackmail awareness in the structural equation model. For descriptive purposes, awareness levels were categorized as inadequate (< 60%), moderate (60–<75%), and high (75–100%).

### Tool 2: Smartphone addiction scale

Kwon et al. created this tool in 2013. A 10-item Likert scale is used. A 6-point Likert scale, with 1 denoting “strongly disagree” and 6 denoting “strongly agree,” is used in this scale. The total score falls between 10 and 60. The range of smartphone addiction is 10˂26.6 for low levels, 16.6 33.2 for moderate levels, and 33.2 60 for high levels. Increased scores signify a high level of smartphone addiction. The SAS had a Cronbach’s alpha correlation coefficient of 0.91 [16]. The corrected item total correlation coefficients fell well within Nunnally & Bernstein’s [17] acceptable range, ranging from 0.50 to 0.80.

### Tool 3: Escapism social scale

Gao et al. (2017) created the Escapism Social Media Scale (ESMS) [18]. The four-item ESMS, which was derived from the Escapism Scale (Hirschman, 1983; updated by Wu & Holsapple, 2014), was used to measure escapism related to social media. On a seven-point rating system, items (e.g., “This SNS helps me escape from the world of reality”) are ranked from 1 (strongly disagree) to 7 (strongly agree). Higher scores suggest greater escapism tied to social media. The overall score goes from 4 to 28.

### Pilot study

A pilot study was conducted with 10% of the nursing students (*n* = 44) to evaluate the tools’ usefulness, clarity, and usability, as well as to identify any potential issues during data collection. Internal consistency and participant feedback were assessed to ensure the reliability and validity of the instruments. The results indicated that all items were clear, relevant, and aligned with the study objectives. Participants reported no difficulties or confusion in understanding the questions. Consequently, no modifications were deemed necessary. To prevent data contamination, the pilot participants were excluded from the main study.

### Data collection

The study instruments were reviewed by expert faculty to ensure cultural and contextual relevance, with minor wording adjustments made for clarity. A pilot test confirmed the reliability and comprehensibility of the adapted tools. During data collection, nursing students received questionnaires directly from the researchers and completed them on-site under supervision, allowing immediate clarification of any queries and ensuring completeness. Students were recruited across clinical units, which facilitated rapid distribution and collection. Each questionnaire required approximately 15–20 min to complete, and data were gathered over a two-month period between July and August 2025.

### Data analysis

After systematic coding and data entry, the dataset was checked for missing values before analysis. The analysis did not include questionnaires with more than 20% missing replies. Mean replacement within the corresponding scale was used to manage missing values for questionnaires with limited missing data (< 5%), which is deemed acceptable when the percentage of missing data is modest and randomly distributed.

Data was analyzed using IBM SPSS Statistics and AMOS version 23. Descriptive statistics (frequencies, percentages, means, and standard deviations) were used to summarize participants’ demographic characteristics and study variables. Pearson correlation analysis and hierarchical multiple linear regression were conducted to examine the relationships among electronic blackmail awareness, smartphone addiction, and escapism.

To examine the underlying factor structure of the study instruments within the target population, Exploratory Factor Analysis (EFA) was performed using the principal component extraction method with Varimax rotation as a preliminary validation step. The suitability of the data for factor analysis was assessed using the Kaiser–Meyer–Olkin (KMO) measure of sampling adequacy and Bartlett’s test of sphericity.

Subsequently, covariance-based Structural Equation Modeling (SEM) using AMOS was conducted to test the hypothesized relationships among electronic blackmail awareness, smartphone addiction, and escapism. Model fit was evaluated using several goodness-of-fit indices, including the chi-square to degrees of freedom ratio (χ²/df), Comparative Fit Index (CFI), Incremental Fit Index (IFI), Normed Fit Index (NFI), Relative Fit Index (RFI), and Root Mean Square Error of Approximation (RMSEA). In addition, convergent and discriminant validity of the constructs were assessed using composite reliability (CR), average variance extracted (AVE), and the Fornell–Larcker criterion. Statistical significance was set at *p* ≤ 0.05.

## Results

### Reliability statistics

Internal consistency reliability was assessed separately for each measurement scale using Cronbach’s alpha. The Smartphone Addiction Scale demonstrated high reliability (α = 0.845), followed by the Escapism Social Scale (α = 0.805) and the Electronic Blackmail Awareness scale (α = 0.753). The overall internal consistency of the combined instrument was acceptable (α = 0.764).

**Table 1:** Reliability statistics for the study tool (*n* = 440)

Dimensions	Cronbach’s Alpha	*N* of Items
Electronic Blackmail Awareness	0.753	13
Smartphone addiction scale	0.845	10
Escapism Social Scale	0.805	4
**Total**	0.764	27

Furthermore, to examine the underlying factor structure of the study instruments within the current sample, exploration factor analysis (EFA) was conducted as a preliminary step. Sampling adequacy was confirmed, with a Kaiser–Meyer–Olkin (KMO) value of 0.816, exceeding the recommended threshold of 0.60. Bartlett’s test of sphericity was statistically significant (χ² = 3138.311, df = 351, *p* < 0.001), indicating that the correlation matrix was suitable for factor extraction (Table 2).

**Table 2:** KMO and Bartlett’s test of sphericity

KMO and Bartlett’s Test		
Kaiser-Meyer-Olkin Measure of Sampling Adequacy.		0.816
Bartlett’s Test of Sphericity	Approx. Chi-Square	3138.311
	df	351
	Sig.	0.000

According to Table 3, every item had appropriate communalities (> 0.40), signifying that each one made a significant contribution to the variables that were retrieved in the Principal Component Analysis.

**Table 3:** Sample communalities of items after extraction

Communalities		
	Initial	Extraction
Awareness_1	1.000	0.507
Awareness_2	1.000	0.424
Awareness_3	1.000	0.642
Awareness_4	1.000	0.440
Awareness_5	1.000	0.602
Awareness_6	1.000	0.510
Awareness_7	1.000	0.454
Awareness_8	1.000	0.512
Awareness_9	1.000	0.657
Awareness_10	1.000	0.699
Awareness_11	1.000	0.649
Awareness_12	1.000	0.646
Awareness_13	1.000	0.573
S1	1.000	0.439
S2	1.000	0.671
S3	1.000	0.621
S4	1.000	0.587
S5	1.000	0.658
S6	1.000	0.643
S7	1.000	0.701
S8	1.000	0.631
S9	1.000	0.535
S10	1.000	0.491
E1	1.000	0.542
E2	1.000	0.757
E3	1.000	0.675
E4	1.000	0.629

Table 4 presents the results of the exploratory factor analysis based on the principal component extraction method. The analysis identified seven components with eigenvalues greater than 1, which together explained 58.13% of the total variance. The first component accounted for 20.39% of the variance, followed by the second component explaining 13.13%, indicating that these components contribute substantially to the underlying structure of the instrument. The remaining components explained progressively smaller proportions of variance but still met the eigenvalue criterion for retention.

**Table 4:** Total variance explained

Component	Initial Eigenvalues			Extraction Sums of Squared Loadings		
	Total	% of Variance	Cumulative %	Total	% of Variance	Cumulative %
1	5.505	20.390	20.390	5.505	20.390	20.390
2	3.545	13.131	33.522	3.545	13.131	33.522
3	1.733	6.419	39.941	1.733	6.419	39.941
4	1.496	5.541	45.482	1.496	5.541	45.482
5	1.284	4.754	50.236	1.284	4.754	50.236
6	1.103	4.084	54.320	1.103	4.084	54.320
7	1.029	3.810	58.130	1.029	3.810	58.130
8	0.980	3.631	61.762			
9	0.965	3.574	65.335			
10	0.873	3.234	68.569			
11	0.846	3.133	71.702			
12	0.782	2.895	74.597			
13	0.723	2.678	77.275			
14	0.715	2.650	79.924			
15	0.663	2.454	82.379			
16	0.611	2.261	84.640			
17	0.554	2.051	86.691			
18	0.529	1.961	88.652			
19	0.473	1.752	90.404			
20	0.446	1.650	92.055			
21	0.441	1.635	93.690			
22	0.411	1.524	95.213			
23	0.343	1.270	96.483			
24	0.327	1.211	97.694			
25	0.298	1.102	98.796			
26	0.246	0.912	99.708			
27	0.079	0.292	100.000			

Extraction Method: Principal Component Analysis

**Fig. 2:**
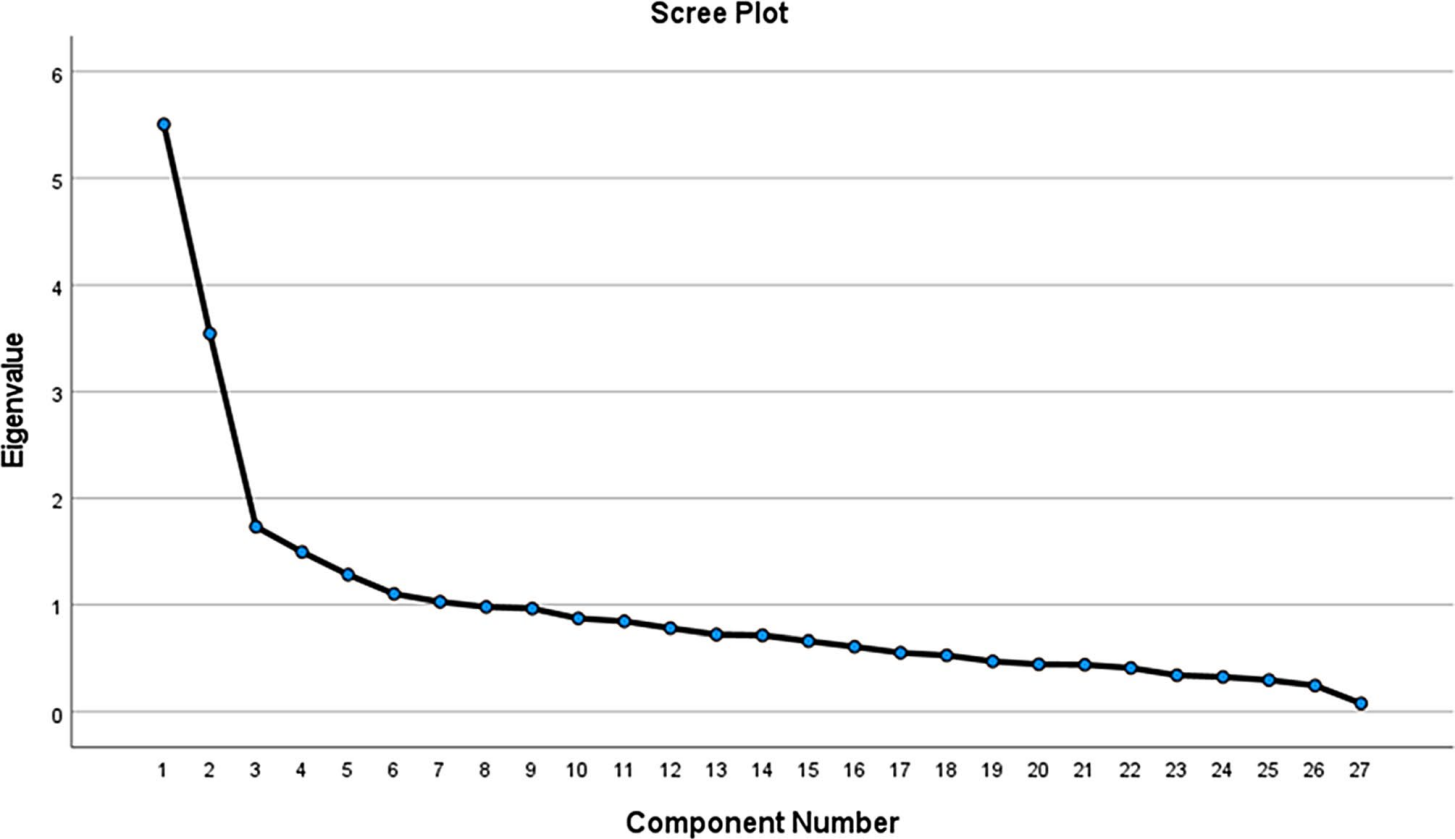
Scree plot showing eigenvalues for principal component analysis

The scree plot illustrates the eigenvalues associated with each component generated during exploratory factor analysis as showed in the Fig. 2. The steep decline followed by a clear inflection after the second component indicates the point at which additional components contribute minimal explanatory variance, supporting retention of the factors above the elbow.

Additionally, Table 5 revealed the exploratory factor analysis identified three principal constructs underlying the study instrument: Smartphone Addiction, Social Media Awareness, and Escapism. All retained items showed factor loading exceeding the acceptable threshold of 0.40, indicating adequate construct representation. Convergent and discriminant validity were evaluated in order to further analyze concept validity. Average variance extracted (AVE) and composite reliability (CR) were used to assess convergent validity. While the AVE values ranged from 0.52 to 0.61, beyond the acceptable value of 0.50, the CR values ranged from 0.84 to 0.88, exceeding the suggested barrier of 0.70. These findings support the constructs’ sufficient convergent validity. The square root of AVE was used to assess discriminant validity; it was found to be larger than the correlations across components, indicating excellent discriminant validity.

**Table 5:** Exploratory factor analysis of the study instrument

Item	Smartphone addiction	Blackmail awareness	Escapism
s4	0.565		
s5	0.724		
s6	0.713		
s7	0.625		
s8	0.627		
s9	0.581		
s10	0.549		
Awareness_Q1		0.603	
Awareness_Q2		0.600	
Awareness_Q3		0.498	
Awareness_Q4		0.555	
Awareness_Q5		0.739	
Awareness_Q6		0.628	
E1			0.520
E2			0.824
E3			0.689
E4			0.567

Table 6 revealed the participants (*N* = 440) had a mean age of 20.57 years (SD = 1.30), according to their demographic profile, representing a group with minimal age variation that is primarily young and developmentally comparable. With 69.8% of the sample being female and 30.2% being male, the study primarily represents the viewpoints of young women, which is in line with the demographics of normal nursing students. Most participants (60.9%) were from rural areas, indicating that their perceptions and behaviors may be more strongly influenced by rural experiences and environments than by urban ones. In terms of financial condition, 51.6% of respondents said their family’s income was insufficient, while 48.4% said it was acceptable, indicating a nearly equal divide in perceived economic stability. This demographic profile shows that the sample is primarily young, female, and rural, with an even distribution of financial adequacy factors that may influence how participants interact with and react to the research variables.

**Table 6:** Distribution of the nurse’s student according to demographic data (*n* = 440)

Demographic characteristics	No.	%
**Age (years)**		
**Mean ± SD =** 20.57 ± 1.30		
**Gender**		
Male	133	30.2
Female	307	69.8
**Place of residence**		
Urban	172	39.1
Countryside	268	60.9
**Family income**		
Enough	213	48.4
Not enough	227	51.6

With 91.8% of participants indicating active interaction on multiple platforms, Table 7 shows a high prevalence of social media use among participants. Just a small percentage of users used PCs (4.5%) or laptops (3.6%) to access social media, but nearly all users (91.8%) performed so through their cell phones. Facebook was the most popular platform (66.8%), followed by TikTok (45.7%), Instagram (34.1%), WhatsApp (31.1%), and Twitter (22.3%), indicating a preference for programs that are visually appealing and socially interactive. In terms of usage time, 34.3% of students reported using social media for about three hours per day, while 28.4% reported using it for four hours or more, showing significant daily exposure. Just a small percentage indicated that they used it very little or not at all.

**Table 7:** Social media usage among nursing students (*N* = 440)

Student usage of social media	No.	%
**Usage of social media**		
Yes	424	91.8
No	16	3.6
**Internet application access**		
Personal Phone	404	91.8
Computer	20	4.5
Laptop	16	3.6
**Common social media platform used**		
Facebook	294	66.8
Tik Tok	201	45.7
WhatsApp	150	34.1
Instagram	137	31.1
Twitter	98	22.3
**Spend hours per day on social media**		
I don’t use it	24	5.5
> Half an hour	52	11.8
Half an hour: an hour	88	20.0
3 h	151	34.3
≥ 4 h	125	28.4
**Good relationship between you and your parents**		
Yes	422	95.9
No	18	4.1
**Monitoring and follow-up from the family when you use social media**		
Yes	247	56.1
No	193	43.9
**Parents discussed with you the dangers of using social media**		
Yes	313	71.1
No	127	28.9
**Faculty conducted multiple educational workshops**		
Yes	237	53.9
No	203	46.1
**Parents and lecturers need awareness about the dangers of social media**		
Yes	360	81.8
No	80	18.2

Different levels of awareness and experience are revealed by the distribution of nurses according to their behavioral tendencies and electronic blackmail awareness, smartphone addiction, and escapism as shown in Table 8. Just 11.8% of students showed high awareness of electronic blackmail, compared to 21.4% who had intermediate awareness and the majority (66.8%) who had little awareness. The nurses’ overall mean score for their electronic blackmail awareness was 24.36 (SD = 6.55), which suggests that they are generally not very aware of it. Conversely, just 6.4% of nurses reported having a low level of smartphone addiction, whereas a significant percentage reported moderate (47.4%) and high (45.2%) levels. A high frequency of addictive smartphone behaviors was suggested by the mean score of 41.10 (SD = 9.96), which corresponded to a mean item score of 4.11 (SD = 0.99). In terms of escapism, 35.0% of individuals indicated high levels, 15.9% reported low levels, and nearly half (49.1%) reported moderate levels. With an item score of 4.59 (SD = 1.38), the overall mean score for escapism was 18.37 (SD = 5.55), suggesting that many nurses have a propensity to use escapist behaviors as a coping strategy. Whenever considered as a whole, these results show an upward trend of low awareness of digital risks but significant participation in technology-related activities, indicating a potential vulnerability that requires focused preventive and instructional measures.

**Table 8:** Distribution of the nurses studied according to their electronic blackmail awareness, smart phone addiction and escapism (*N* = 440)

	Low		Moderate		High		Total score	Mean score
	No.	%	No.	%	No.	%	Mean ± SD	Mean ± SD
Electronic Blackmail Awareness	294	66.8	94	21.4	52	11.8	24.36 ± 6.55	18.69 ± 8.02
Smartphone addiction	28	6.4	209	47.4	199	45.2	41.10 ± 9.96	4.11 ± 0.99
Escapism	70	15.9	216	49.1	154	35.0	18.37 ± 5.55	4.59 ± 1.38

Table 9 The predictors of escapism were investigated using hierarchical multiple regression analysis. Awareness of electronic blackmail did not substantially predict escapism in Model 1 (B = 0.018, β = 0.024, *p* = 0.576). Model 2 explained 29.5% of the variance in escapism after integrating smartphone addiction (R² = 0.295, Adjusted R² = 0.293). While awareness of electronic blackmail was not significant (B = 0.015, β = 0.019, *p* = 0.583), smartphone addiction was found to be a substantial and significant predictor of escapism (B = 0.302, β = 0.543, *p* < 0.001).

**Table 9:** Hierarchical regression analysis predicting escapism (*N* = 440)

Model		Unstandardized Coefficients		Standardized Coefficients	t	Sig.	R square	Adjusted R square	95.0% Confidence Interval for B	
		B	Std. Error	Beta					Lower Bound	Upper Bound
1	(Constant)	18.068	0.518	0.024	34.858	0.000	0.001	-0.001	17.050	19.086
	Blackmail awareness	0.018	0.032		0.559	0.576			− 0.045	0.080
2	(Constant)	5.690	0.918		6.197	0.000	0.295	0.293	3.886	7.493
	Blackmail awareness	0.015	0.027	0.019	0.549	0.583			− 0.038	0.067
	Smartphone addiction	0.302	0.020	0.543	15.313	0.000			0.264	0.341

R^2^: Coefficient of determination

B: Unstandardized Coefficients

Beta: Standardized Coefficients

t: t-test of significance

CI: Confidence interval

LL: Lower limit

UL: Upper Limit

*: Statistically significant at *p* ≤ 0.05

**Fig. 3:**
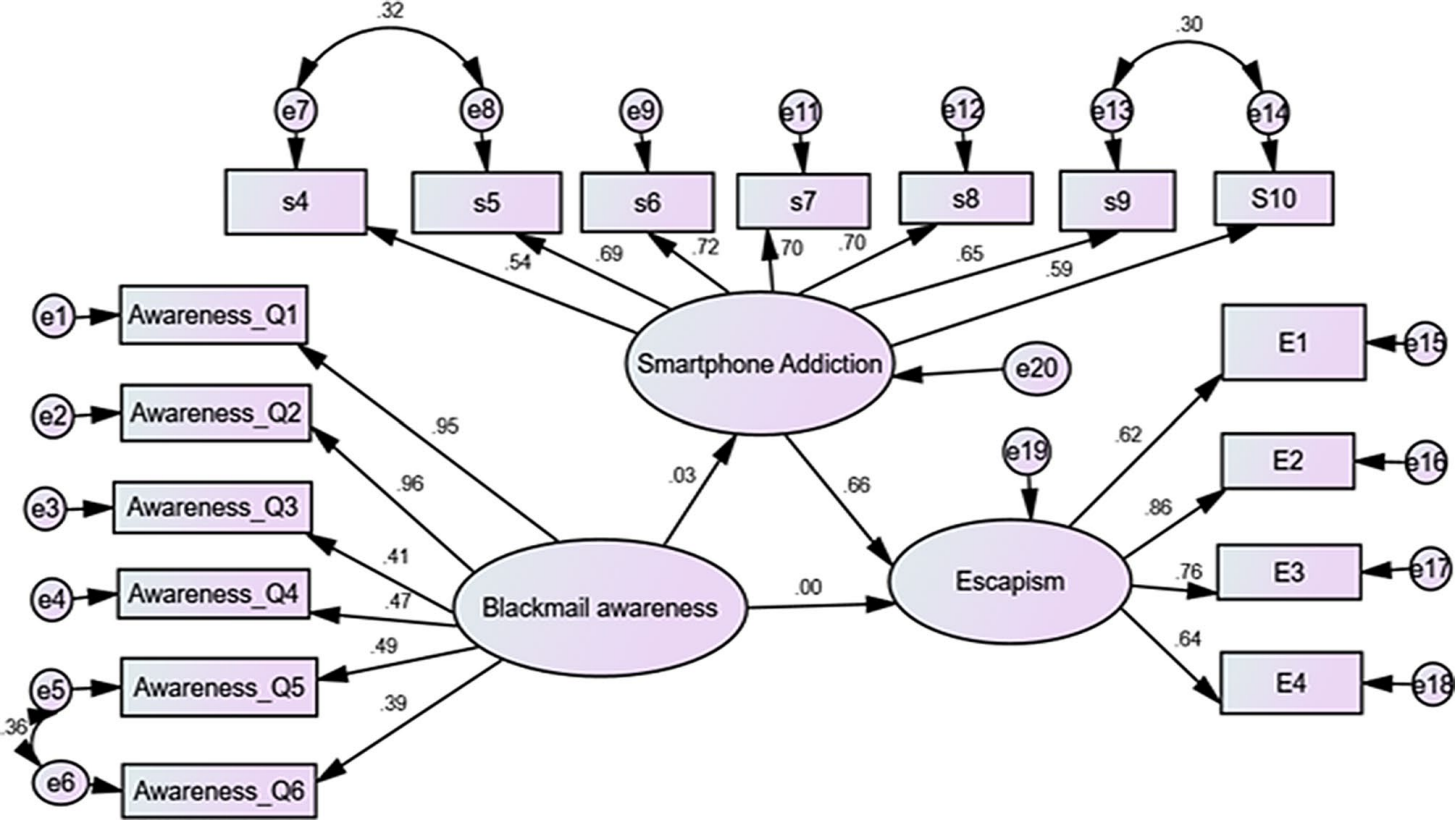
Structural equation model illustrating the relationships among electronic blackmail awareness, smartphone addiction, and escapism among nursing students

Table 10 revealed the associations between nursing students’ awareness of electronic blackmail, smartphone addiction, and escapism were investigated using structural equation modeling (SEM) also showed in Fig. 3. Although structural paths are directed by model specification, all path estimates are supposed to be associations rather than causal effects due to the cross-sectional character of this study. The findings demonstrated that smartphone addiction was not substantially associated with awareness of electronic blackmail (B = 0.035, β = 0.034, SE = 0.048, CR = 0.725, *p* = 0.469). Similarly, escapism was not significantly associated with awareness of electronic blackmail (B = 0.001, β = 0.001, SE = 0.039, CR = − 0.005, *p* = 0.996). On the other hand, escapism was strongly and statistically significantly positively associated with smartphone addiction (B = 0.645, β = 0.664, SE = 0.061, CR = 10.630, *p* < 0.001). These results show that while awareness of electronic blackmail was not significantly associated with smartphone addiction or escapism, higher levels of smartphone addiction are linked to increased escapist behaviors among nursing students. The structural model showed acceptable goodness-of-fit indices in terms of model fit. A satisfactory model fit was indicated by the chi-square to degrees of freedom ratio of χ²/df = 3.112 (*p* < 0.001). Other fit indices, such as NFI = 0.918, RFI = 0.901, IFI = 0.943, and CFI = 0.942, all of which were higher than the suggested cutoff of 0.90, confirmed the model’s suitability. Additionally, an acceptable degree of approximation error was indicated by the Root Mean Square Error of Approximation (RMSEA), which was 0.061. Overall, these findings imply that the suggested structural model adequately captures the connections between smartphone addiction, escapism, and awareness of electronic blackmail.

**Table 10:** Structural path estimates of the SEM model (*N* = 440)

Path	B (Estimate)	Beta (Standardized)	S.E.	C.*R*.	*P*
Smartphone addiction ← Electronic Blackmail Awareness	0.035	0.034	0.048	0.725	0.469
Escapism ← Electronic Blackmail Awareness	0.001	0.001	0.039	-0.005	0.996
Escapism ← Smartphone addiction	0.645	0.664	0.061	10.630	< 0.001

Model fit parameters showed acceptable goodness of fit, with NFI = 0.918, RFI = 0.901, IFI = 0.943, and CFI = 0.942. The RMSEA was 0.061, indicating an acceptable approximation error. The chi-square to degrees of freedom ratio (χ²/df) was 3.112 (*p* ≤ 0.001), suggesting an acceptable model fit

**CFI**: Comparative Fit Index

**IFI**: Incremental Fit Index

**RMSEA**: Root Mean Square Error of Approximation

## Discussion

This study examined the relationships among electronic blackmail awareness, smartphone addiction, and escapism among nursing students. Consistent with previous research, participants reported high levels of social media engagement, with Facebook being the most frequently used platform, followed by TikTok and Instagram [19–21]. These findings align with earlier studies showing that social media plays a central role in students’ daily communication, entertainment, and academic activities, although platform preferences may vary across contexts [22].

Nearly one-third of participants reported spending four hours or more per day on social media, reflecting extensive digital exposure. Similar patterns have been documented among adolescents and university students, where excessive social media use is often associated with limited parental monitoring and insufficient awareness of digital risks [23]. Together with the observed gaps in digital safety knowledge, these findings highlight the importance of comprehensive digital literacy and family-centered interventions. Previous research has emphasized integrating digital safety education into academic curricula and strengthening digital parenting practices to reduce online vulnerability [24–28].

A key finding of this study was that most participants demonstrated inadequate knowledge of electronic blackmail. This limited awareness may be related to insufficient institutional awareness programs, the rapid evolution of digital technologies, and sociocultural barriers surrounding cyber-extortion [3, 29–31]. At the same time, relatively high levels of smartphone addiction and escapism were observed among the participants. These findings are consistent with previous research linking excessive smartphone use to psychological distress, academic pressure, and increased reliance on digital platforms as a coping or avoidance strategy [32–36].

The structural model results indicated that awareness of electronic blackmail was not significantly associated with either smartphone addiction or escapism. This finding suggests that awareness of cyber risks may primarily represent a cognitive understanding of online threats rather than a factor directly related to behavioral coping responses. One possible explanation is that exposure to cyber-risk information may increase knowledge and risk recognition but does not necessarily generate emotional responses strong enough to be reflected in behavioral outcomes such as excessive smartphone use or escapism [37, 38].

In contrast, smartphone addiction was significantly and positively associated with escapism. This finding is consistent with theoretical perspectives such as the Interaction of Person–Affect–Cognition–Execution (I-PACE) model, which conceptualizes problematic technology use as related to emotional regulation processes, reward sensitivity, and reduced self-control [39, 40]. Within this framework, excessive smartphone use may serve as a mechanism through which individuals temporarily disengage from stress or negative emotions, thereby contributing to escapist tendencies.

Overall, the findings indicate that escapism among nursing students appears to be more strongly associated with behavioral patterns related to smartphone addiction than with awareness of electronic blackmail alone. These findings have important implications for intervention strategies. Awareness programs focusing solely on cyber threats may not be sufficient to address problematic technology-related behaviors. Instead, prevention efforts may benefit from integrating digital wellness education, self-regulation strategies, and psychosocial support within nursing education programs. Such multidimensional approaches may help promote healthier digital habits and improve students’ psychological well-being.

## Conclusion

This study indicates that nursing students’ escapist tendencies are more strongly associated with patterns of compulsive smartphone use than with their awareness of electronic blackmail. Rather than functioning as a direct behavioral factor, cyber-awareness appears insufficient on its own to relate to students’ coping responses, highlighting a gap between recognizing online risks and translating this knowledge into protective digital practices. The findings also suggest that smartphone addiction plays a more prominent role in students’ technology-related behavioral patterns than awareness of cyber threats. These results contribute to the growing literature on digital behavior and nursing education by emphasizing that cognitive understanding of cyber risks may not necessarily correspond with behavioral outcomes.

From a practical perspective, the findings highlight the importance of addressing problematic smartphone use within nursing education. Interventions aimed at promoting digital health among nursing students may benefit from extending beyond awareness campaigns to include structured digital-wellness education, strategies to encourage responsible smartphone use, and training that supports adaptive coping skills. Such approaches may help foster healthier digital habits and support students’ psychological well-being, academic functioning, and readiness for future professional roles.

### Strengths and limitations

This study offers insights into the relationships among electronic blackmail awareness, smartphone addiction, and psychological escapism among nursing students. Several strengths should be acknowledged, including the use of validated measurement instruments, a proportionally stratified sampling approach, and the application of structural equation modeling to examine the relationships among the study variables. However, several limitations should also be considered. First, the cross-sectional design prevents the establishment of causal relationships among the variables, and the findings should therefore be interpreted as associations rather than causal effects. Second, the data were collected through self-reported questionnaires, which may introduce response biases such as recall bias or social desirability bias. Third, the sample was drawn from a single academic institution, which may limit the generalizability of the findings to nursing students in other educational or cultural contexts.

Additionally, electronic blackmail awareness was measured using a knowledge-based assessment, which primarily captures cognitive understanding of cyber risks and may not fully represent broader aspects of awareness such as attitudes, perceived vulnerability, or behavioral readiness. Future research may benefit from employing longitudinal and multi-site study designs, incorporating objective or mixed-method measures, and testing more complex structural models to further examine the relationships among cyber-risk awareness, technology-related behaviors, and coping strategies among nursing students.

### Implications for nursing practice

The findings highlight important considerations for clinical settings. Nursing students often encounter academic and clinical demands that may increase their reliance on maladaptive coping strategies, including excessive smartphone use. Such patterns can undermine concentration, clinical judgement, and patient-safety competencies. Incorporating digital wellness principles into clinical training may help students develop healthier coping behaviors and maintain situational awareness during patient care. Clinical instructors and preceptors also have a key role in monitoring students’ digital habits, modelling balanced technology use, and offering timely guidance to support professional behavior in clinical environments. However, because the study was conducted in a single university in Egypt, these implications should be interpreted within similar educational and cultural contexts and may require adaptation before application in other settings.

### Implications for nursing education and policy

In educational contexts, the results emphasize the need to embed structured content on digital literacy, healthy technology engagement, and strategies to prevent maladaptive digital coping. Cyber-awareness initiatives addressing electronic blackmail should be supported by resilience-building components including stress-management, mindfulness, or cognitive-behavioral approaches to ensure that awareness is translated into safe and adaptive online practices. At a policy level, institutions should develop clear guidelines for digital safety, provide access to counselling for cyber-related concerns, and maintain accessible reporting mechanisms for online threats. Integrating digital health and psychosocial safety principles into nursing curricula may strengthen students’ academic engagement, clinical readiness, and long-term well-being.

### Recommendations for future research

Future studies should adopt longitudinal or multi-center designs to improve generalizability and clarify causal pathways among electronic blackmail awareness, smartphone addiction, and escapism. Building on the limitations of this study, mixed-methods approaches may provide deeper insight into the contextual factors shaping students’ digital coping behaviors. Investigating potential moderate variables such as gender, socioeconomic context, or levels of social support may further refine understanding of these relationships. Intervention-based research is also needed to evaluate the effectiveness of strategies such as mindfulness training, digital detox programs, or faculty-supported behavioral coaching in reducing smartphone addiction and escapist tendencies. Cultural comparisons may offer additional insight into how perceptions of cyberthreats and coping strategies differ across educational and healthcare environments. Given the single-institution design of this study, these recommendations should be considered context-sensitive and may need modification when applied to different educational systems or cultural environments.

## Electronic Supplementary Material

Below is the link to the electronic supplementary material.

Supplementary Material 1

## Data Availability

The datasets generated during and analyzed during the current study are available from the corresponding author upon reasonable request.
