# Supplementary material for: Exploring the relationships between electronic blackmail awareness, smartphone addiction, and escapism among nursing students: a structural equation modeling approach
Source: BMC Nurs. 2026 May 11;25:436. doi: 10.1186/s12912-026-04710-z (PMC13162359; doi:10.1186/s12912-026-04710-z)
Supplement: Supplementary file 1 — Supplementary Material 1 [file 12912_2026_4710_MOESM1_ESM.docx]

**Composite… Reliability, Average Variance Extracted, and Discriminant Validity of the Study Constructs**

| **Construct** | **Cronbach’s α** | **CR** | **AVE** | **√AVE** |
| --- | --- | --- | --- | --- |
| Electronic Blackmail Awareness | 0.753 | 0.84 | 0.52 | 0.72 |
| Smartphone Addiction | 0.845 | 0.88 | 0.55 | 0.74 |
| Escapism | 0.805 | 0.86 | 0.61 | 0.78 |
|  |  |  |  |  |

**Table …Discriminant Validity Using the Fornell–Larcker Criterion**

| **Construct** | **Electronic Blackmail Awareness** | **Smartphone Addiction** | **Escapism** |
| --- | --- | --- | --- |
| Electronic Blackmail Awareness | **0.72** |  |  |
| Smartphone Addiction | 0.17 | **0.74** |  |
| Escapism | 0.16 | 0.53 | **0.78** |
